# National HBsAb serosurvey and HBsAg/Hepatitis D coinfection prevalence across sociodemographic and immigrant populations in Israel

**DOI:** 10.1186/s12879-026-14403-2

**Published:** 2026-09-12

**Authors:** Gozlan Yael, Asraf Keren, Keinan-Boker Lital, Arami S. Shoshana, Picard Orit, Mor Orna, Bassal Ravit

**Affiliations:** 1 Central Virology Laboratory, Public Health Services, Ministry of Health, Chaim Sheba Medical Center, Ramat-Gan, Israel; 2 The Dworman Automated-Mega Laboratory, Sheba Medical Center, Tel Hashomer, Ramat- Gan, Israel; 3 Israel Center for Disease Control, Ministry of Health, Sheba Medical Center, Ramat-Gan, Israel; 4 School of Public Health, University of Haifa, Haifa, Israel; 5 Gastroenterology Laboratory, Sheba Medical Center, Ramat Gan, 52621 Israel; 6 Department of Epidemiology and Preventive Medicine, School of Public Health, Gray Faculty of Medical and Health Sciences, Tel Aviv University, Tel Aviv, Israel

**Keywords:** Hepatitis B, Hepatitis D, Seroprevalence, HBsAb, HBsAg, High risk groups

## Abstract

**Background:**

Hepatitis B virus (HBV) remains a global health burden despite widespread vaccination efforts. This nationwide serosurvey aimed to assess Hepatitis B surface antibody (HBsAb) seropositivity in Israel and HBV/ Hepatitis D virus (HDV) prevalence among immigrant populations, in the context of universal HBV vaccination introduced nationally in 1992.

**Methods:**

A representative sample of 2,423 individuals was collected in 2023, and screened for HBsAb. Hepatitis B surface antigen (HBsAg) prevalence was assessed in additional 1,000 samples of selected immigrant populations, and positive samples were screened for HDV. Data were analyzed by age, sex, ethnicity, and socioeconomic status.

**Results:**

Overall HBsAb seropositivity was 42.8% (95% CI: 40.9–44.8%). HBsAb seropositivity was significantly higher in individuals born after in or after 1992 compared to those born before (49.2% vs. 36.0%, *p* < 0.0001). Significant disparities were observed: lower socioeconomic status and peripheral residency were associated with lower HBsAb seropositivity (compared to high SES: OR = 1.51, 95% CI: 1.29–1.78; and Central residency: OR = 1.61, 95% CI: 1.23–2.09). Ethnic differences were prominent, with Jews and other minorities population showing higher rates in younger cohorts, while Arab population had higher rates in older populations. HBsAg prevalence was high (9%) among immigrants from Ethiopia as well as from Ukraine, with HDV coinfection detection in 8.2% of HBsAg carriers from Africa.

**Conclusions:**

While vaccination program has been effective, significant HBsAb and HBsAg seroprevalence gaps persist in the adult population and specific socio-demographic groups., Screening strategies should be considered, particularly for high-risk immigrants and specific immigrant populations.

**Supplementary Information:**

The online version contains supplementary material available at https://doi.org/10.1186/s12879-026-14403-2.

## Introduction

Hepatitis B virus (HBV) remains a significant global health challenge, affecting approximately 296 million people worldwide. Each year, HBV causes over 800,000 deaths, primarily due to severe liver-related complications such as cirrhosis and hepatocellular carcinoma (HCC). Chronic HBV infection is a leading cause of long-term morbidity and mortality, posing a substantial economic burden on healthcare systems worldwide [1]. Furthermore, hepatitis D virus (HDV) coinfection among chronic HBV carriers represents an additional challenge, potentially accelerating liver disease progression despite low active HBV replication rates [2]. Therefore, the World Health Organization (WHO) has set 2030 as a target year for elimination of HBV as a public health threat, aiming to reduce the rate of new infections and of HBV-related mortality by 90% and 65%, respectively [3].

Vaccination has been one of the most effective and safe measures to prevent HBV (and therefore also HDV), leading to significant reductions in acute infection and chronic carrier rates in vaccinated populations [4]. Nevertheless, certain populations remain particularly vulnerable to hepatitis B virus infection, including infants, migrants and refugees from endemic regions, people who inject drugs, individuals with high-risk sexual behaviors (including men who have sex with men), people experiencing homelessness, and those with low socioeconomic status [5]. These groups face increased risk due to factors such as perinatal transmission, unsafe injections, limited access to healthcare, and social exclusion.

In Israel, HBV vaccination was successfully introduced into the national immunization program in 1992, with a three-dose schedule administered at birth, one month, and six months of age [6]. A previous study conducted on samples collected in 2011 demonstrated that 49% of vaccinated children and adolescents were anti-HBs seropositive and found no significant differences by gender, ethnicity (Jews and other minorities population vs. Arab population), or country of birth (Israel vs. abroad) [7]. However, in that study, seroprevalence was not assessed for individuals born before 1992. As demonstrated previously, individuals born before 1992 in Africa or in Eastern Europe, as well as the Israeli Arab population, were at higher risk of past exposure to HBV [8–11].

The aim of this study was to estimate the prevalence of antibodies to HBV in the Israeli population in 2023 and to determine the prevalence of those infected with HBV among selected immigrant populations born before 1992. In addition, we aimed to assess HDV seroprevalence and viremia among the HBsAg-positive high-risk individuals.

## Materials and methods

### Study population

All samples used in this study were retrieved from the Israel Center for Disease Control (ICDC) sera bank, which keeps a collection of residual blood specimens that represent the Israeli population by age, gender, ethnicity (Jews and other minorities population /Arab population) and geographic district [12]. Comparison of the study sample distribution and the Israeli general population by age group, sex, and population group is presented in Supplementary Table 1. The samples were residual sera collected from individuals who underwent routine blood testing at community clinics and hospital outpatient settings across Israel, and were not specifically collected from ill patients. Accordingly, 2,423 samples collected in 2023 were selected. These samples were selected to achieve a distribution representative of the Israeli population with respect to age, sex, and geographic region, based on population data from the Israeli Central Bureau of Statistics (CBS). Specifically, a stratified sampling approach was applied, in which strata were defined by age group, sex, and district of residence. Within each stratum, samples were selected randomly from all available residual specimens meeting the inclusion criteria. To assess the incidence of HBV infection in those born before 1992, considered to be at risk for being HBsAg positive, based on estimated prevalence of 3–6% [6, 11] and power of 80%, additional 1,000 samples were collected between 2020 and 2023 from Jewish individuals born in Africa, Eastern Europe, Yemen, and Arab individuals. Countries of origin were grouped to reflect known differences in HBV endemicity; Yemen, Morocco, Ethiopia, Ukraine and Russia were specifically represented due to substantially varying HBV prevalence in these regions, while individuals from other countries of origin were included under broader regional groupings. Data collected for each sample were age, sex, ethnic group, birth country, district of residence and socioeconomic index. The Israeli Central Bureau of Statistics (CBS) assigns a socioeconomic index ranging from 1 to 10, where 1 represents the lowest socioeconomic status and 10 the highest. The index is calculated at the locality or statistical-area level and is based on a composite of demographic, educational, employment, and standard-of-living indicators [13]. The study protocol was approved by the institutional review board of the Sheba Medical Center (1378-24-SMC), and a waiver of informed consent was granted.

### Laboratory analysis

The presence of HBsAb in serum was measured using HBsAb kit on the Alinity instrument (Abbott). This test was reported to have 98.3% sensitivity and 100.0% specificity compared to other commercially available HBsAb ELISA tests. The presence of HBsAg was tested on Alinity using HBsAg Qualitative II assay kit (Abbott), with reported sensitivity of 89.4% and specificity of 98.1% [14]. All assays were performed according to the manufacturer’s instructions. For HBsAb, the quantitative results were categorized dichotomously, with a titer of 10 IU/mL defined as positive, representing the internationally recognized threshold for protective immunity. HBsAg and HDV Ab results were interpreted qualitatively as reactive or non-reactive based on the manufacturer’s established signal-to-cutoff (S/CO) thresholds.

HDV Ab were measured using Liaison XL anti HDV (DiaSorin). HDV viral loads were assessed using EuroBioplex (EuroBio, France, LOD 100 IU/mL). Laboratory analyses were conducted at the Megalab (HBV assays) or the Gastroenterology Laboratory (HDV assay) of the Sheba Medical Center, a fully accredited clinical diagnostic facility. Testing for HBsAb, HBsAg and HDV Ab was performed using validated, routine commercial assays in accordance with the manufacturer’s instructions, incorporating standard internal quality controls (QC) and routine calibration procedures.

### Statistical analysis

HBV seropositivity rates, along with 95% confidence interval (CIs), were calculated and reported as percentages. Differences in the prevalence of HBV seropositivity within subpopulations were examined using the chi-square test and Fisher exact test where appropriate for categorical variables. Logistic regression models were applied to examine associations between demographic characteristics and HBsAb seropositivity, yielding odds ratios (OR) and corresponding 95% CIs. Independent variables included in the model were: age group (or birth cohort), sex, population group (Jews and other minorities / Arab population), district of residence, country of origin, and socioeconomic index. To assess whether the association between birth cohort and HBsAb seropositivity differed by sex, a cross-product interaction term **(sex × birth cohort)** was included in the model based on the a priori hypothesis regarding differential vaccine uptake. Two-sided p-value < 0.05 was considered statistically significant. Statistical analysis was performed using SAS Enterprise Guide (version 8.5, SAS Institute Inc., Cary, NC, USA).

## Results

A total of 2,423 samples, from the National Sera Bank which generally reflect the demographic distribution of the Israeli population in terms of age, sex, and region, were used for assessing the prevalence of HBsAb in Israel, using the Chi-square test (Table 1).

**Table 1:** Demographic characteristics of 2423 samples tested for HBsAb seroprevalence

Characteristics		*n* (%)
Age (years)	0.5>	(1.6) 39
	0.5–0.9	(0.9) 22
	1–4	(7.1) 173
	5–14	(18.2) 440
	15–19	(8.1) 196
	20–24	176 (7.3)
	25–29	161 (6.6)
	30–34	164 (6.8)
	35–44	310 (12.8)
	45–54	263 (10.8)
	55–64	188 (7.8)
	65–74	163 (6.7)
	75+	128 (5.3)
Sex	Male	1216 (50.2)
	Female	1207 (49.8)
Ethnic group	Jews and other minorities population	1966 (81.1)
	Arab population	457 (18.9)
Birth country	Israel	2037 (84.1)
	Other	384 (15.9)
District of residence	Jerusalem	302 (12.5)
	North	400 (16.5)
	Haifa	271 (11.2)
	Central	530 (21.9)
	Tel-Aviv	423 (17.5)
	South	370 (15.3)
	Judea and Samaria	127 (5.2)
Socioeconomic index	1–5	1280 (53.3)
	6–10	1121 (46.7)

Differences in HBsAb seropositivity rates between groups were assessed using the Chi-square test

**Fig. 1:**
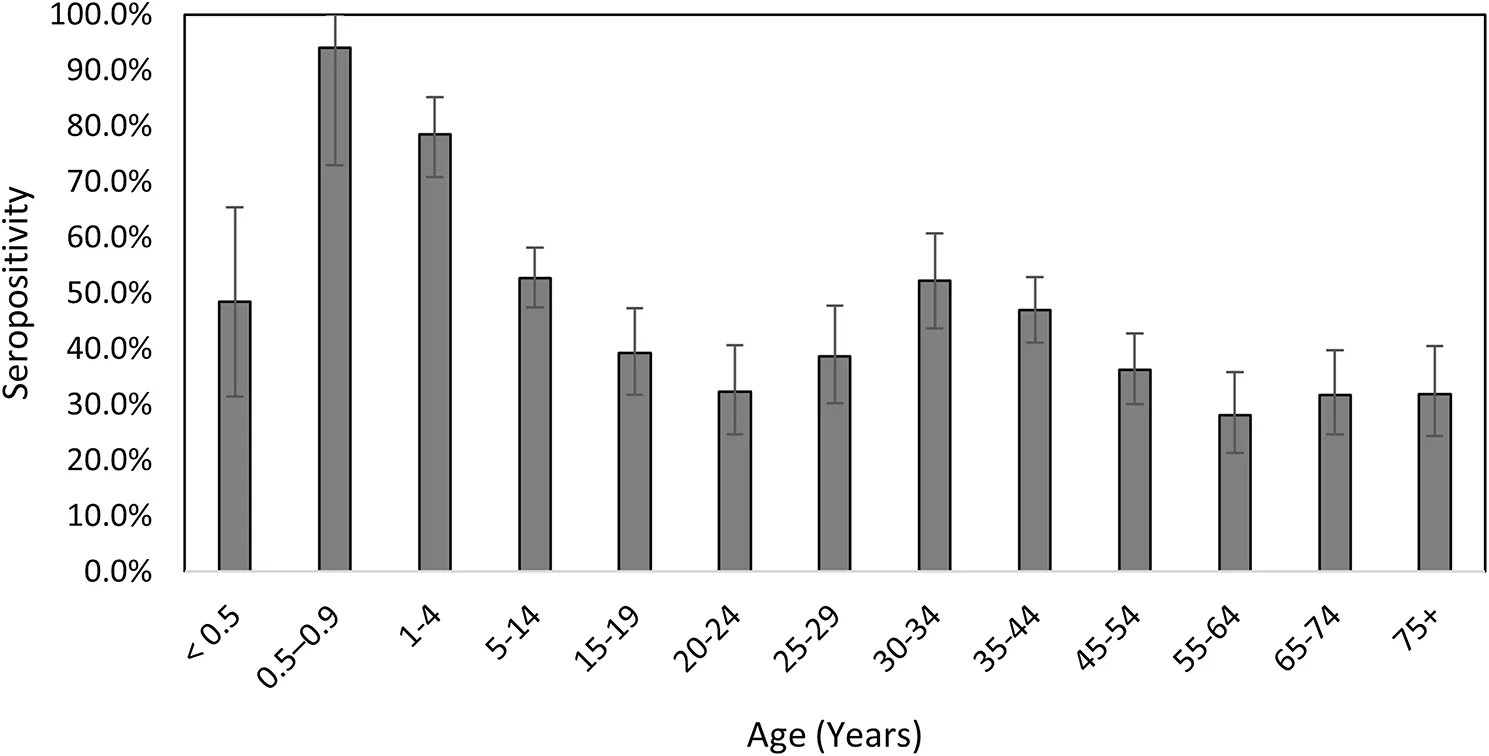
HBsAb seropositivity by age group. Error bars represent 95% confidence intervals. Differences across age categories were assessed using the Chi-square test (*p* < 0.0001)

Overall, 1038 (42.8%; 95% CI: 40.9–44.8%) were HBsAb positive (Table 2). The distribution of HBsAb seropositivity rate by age group (Fig. 1) showed HBsAb seropositivity rate of 48.5% in infants younger than 6 months which increased sharply to 94.1% in the 6–12 (0.5–0.9 years) months age group. The HBsAb seropositivity declined with increasing age, dropping to 79.8% in first to four years group and further decreased to 52.8% in the 5–14 years group. This downward trend continued into the adolescent and adult age groups, with HBsAb seropositivity rates of 39.3%, 32.3%, and 38.7% observed in the 15–19, 20–24, and 25-29-years age groups, respectively. Interestingly, a slight increase to 52.3% was noted in the 30–34 years group before declining again in older populations. The age groups 45 to 75 years and older showed consistently lower seropositive rates, ranging from 28.1% to 36.2%. HBsAb seropositivity differed significantly across age categories (Chi-square test, *p* < 0.0001), with the highest rates observed in individuals born after 1992, consistent with the introduction of universal HBV vaccination.

Overall, HBsAb seropositivity was significantly lower among individuals born before 1992 (36.0%) compared to those born in or after 1992 (49.2%; *p* < 0.0001). These rates also differed significantly across various demographic and socioeconomic groups (Table 2). Among females, HBsAb seropositivity was significantly higher for individuals born in or after 1992 (45.4%) compared to those born before 1992 (37.9%, p-value = 0.0082). A similar but more pronounced difference was observed in males, where HBsAb seropositivity was higher (52.7%) in the born-in-or-after-1992 group compared to the born-before-1992 group (34.1%, *p* < 0.0001). Jews and other minorities population showed higher rates of HBsAb seropositivity (44.0%) compared to Arab population (37.9%) (*p* = 0.0168), especially among individuals born before 1992 (37.9% vs. 25.2%, *p* = 0.0013). Geographic disparities were also observed, particularly in Northern Israel where HBsAb seropositivity increased from 29.5% (born before 1992) to 47.2% (born in or after 1992, *p* = 0.0003), and in Southern Israel where rates increased from 30.5% to 50.5% (*p* = 0.0001) in the same groups. Furthermore, socioeconomic status (SES) was a strongly associated with individuals from lower SES (scores 1–5) showing significantly lower HBsAb seropositivity rates (38.0%) compared to those from higher SES (scores 6–10, 48.2%), (*p* < 0.0001).

**Table 2:** HBsAb seropositivity rates by demographic and socioeconomic characteristics, among those who born before and in or after 1992

Category group		Total			Born before 1992			Born in or after 1992			Born before 1992 vs. born in or after 1992
		*N* Positive	Positive % (95% CI)	*p*-value	*N* Positive	Positive % (95% CI)	*p*-value	*N* Positive	Positive % (95% CI)	*p*-value	*p*-value
Sex	Female	503	41.7 (38.9–44.5)	0.2479	227	37.9 (34.0-41.9)	0.1762	276	45.4 (41.4–49.4)	0.0096	0.0082
	Male	535	44.0 (41.2–46.8)		194	34.1 (30.2–38.2)		341	52.7 (48.8–56.6)		< 0.0001
Ethnic group	Jews and other minorities population	865	44.0 (41.8–46.2)	0.0168	378	37.9 (34.9–41.0)	0.0013	487	50.3 (47.1–53.4)	0.1533	< 0.0001
	Arab population	173	37.9 (33.4–42.5)		43	25.2 (18.8–32.3)		130	45.4 (39.6–51.4)		< 0.0001
Birth country	Israel	873	42.9 (40.7–45.0)	0.9677	286	34.5 (31.2–37.8)	0.0655	587	48.6 (45.8–51.5)	0.0595	< 0.0001
	Other	165	43.0 (38.0-48.1)		135	40.2 (34.9–45.6)		30	62.5 (47.4–76.0)		0.0035
District of residence	Northern Israel	152	38.0 (33.2–43.0)	0.0045	61	29.5 (23.4–36.2)	0.0021	91	47.2 (39.9–54.4)	0.2778	0.0003
	Haifa	107	39.5 (33.6–45.6)		52	35.6 (27.9–44.0)		55	44.0 (35.1–53.2)		0.1593
	Central Israel	263	49.6 (45.3–54.0)		109	45.6 (39.2–52.2)		154	52.9 (47.0-58.8)		0.0938
	Jerusalem	116	38.4 (32.9–44.2)		37	31.4 (23.1–40.5)		79	42.9 (35.7–50.4)		0.0435
	Tel-Aviv	192	45.4 (40.6–50.3)		102	40.2 (34.1–46.5)		90	53.2 (45.4–61.0)		0.0080
	Judea and Samaria	54	42.5 (33.8–51.6)		10	25.0 (12.7–41.2)		44	50.6 (39.6–61.5)		0.0068
	Southern Israel	154	41.6 (36.6–46.8)		50	30.5 (23.6–38.2)		104	50.5 (43.4–57.5)		0.0001
Socioeconomic status	1–5	487	38.0 (35.4–40.8)	< 0.0001	156	26.7 (23.2–30.5)	< 0.0001	331	47.6 (43.8–51.3)	0.2151	< 0.0001
	6–10	540	48.2 (45.2–51.1)		264	45.4 (41.3–49.6)		276	51.1 (46.8–55.4)		0.0575
Total		1038	42.8 (40.9–44.8)		421	36.0 (33.3–38.9)		617	49.2 (46.4–51.9)		< 0.0001

Odds ratios (OR) for having HBsAb were calculated using multivariable analysis (Table 3). Specifically, males born after 1992 showed higher vaccine-mediated immunity compared to females born in the same period (OR = 1.34, 95% CI = 1.07–1.67, *p* = 0.0097), while this difference in HBsAb seropositivity was not apparent in those born before 1992. Being Jewish was significantly associated with having HBsAb in the total group (OR = 1.29, 95% CI = 1.05–1.59, *p* = 0.0170) and for those born before 1992 (OR = 1.82, 95% CI = 1.26–2.63, *p* = 0.0015), whereas no significant association was observed among those born after 1992. Analysis of district of residence demonstrated that living in the Central district was strongly associated with having HBsAb in the total group (OR = 1.61, 95% CI = 1.23–2.09, *p* = 0.0004) and among those born before 1992 (OR = 2.01, 95% CI = 1.36–2.97, *p* = 0.0005). No significant association was found among individuals born after 1992. Similarly, residing in Tel-Aviv was associated with having HBsAb for the total group (OR = 1.36, 95% CI = 1.03–1.79, *p* = 0.0319) and among those born before 1992 (OR = 1.61, 95% CI = 1.09–2.37, *p* = 0.0173), while no association was noted for the other districts. Finally, individuals with a high socioeconomic status (SES, scale 6–10) were significantly more likely to have HBsAb across the total group (OR = 1.51, 95% CI = 1.29–1.78 (*p* < 0.0001) and among those born before 1992 (OR = 2.28, 95% CI = 1.79–2.92 (*p* < 0.0001).

**Table 3:** Odds ratios for having HBsAb across sociodemographic and geographic categories

		Total			Born before 1992			Born after 1992		
**Category group**		Odds Ratio	95% CI	p-value	Odds Ratio	95% CI	p-value	Odds Ratio	95% CI	p-value
Sex	Male	1.10	0.94–1.29	0.2481	0.85	0.67–1.08	0.18	1.34	1.07–1.67	0.0097
	Female	Ref.			Ref.			Ref.		
Ethnic group	Jews and other minorities population	1.29	1.05–1.59	0.0170	1.82	1.26–2.63	0.0015	1.21	0.93–1.58	0.1537
	Arab population	Ref.			Ref.			Ref.		
Birth country	Israel	0.99	0.80–1.24	0.9677	0.78	0.60–1.02	0.0658	0.57	0.31–1.03	0.0626
	Other	Ref.			Ref.			Ref.		
District of residence	Central	1.61	1.23–2.09	0.0004	2.01	1.36–2.97	0.0005	1.26	0.88–1.81	0.2140
	Haifa	1.06	0.78–1.46	0.6986	1.32	0.84–2.08	0.2233	0.88	0.56–1.38	0.5820
	Jerusalem	1.02	0.75–1.38	0.9117	1.09	0.67–1.78	0.7216	0.84	0.56–1.27	0.4111
	Judea and Samaria	1.21	0.80–1.81	0.3635	0.80	0.37–1.73	0.5689	1.15	0.69–1.90	0.5957
	South	1.16	0.87–1.55	0.3050	1.05	0.67–1.64	0.8315	1.14	0.77–1.69	0.5055
	Tel-Aviv	1.36	1.03–1.79	0.0319	1.61	1.09–2.37	0.0173	1.28	0.84–1.93	0.2468
	North	Ref.			Ref.			Ref.		
Socioeconomic status	6–10	1.51	1.29–1.78	< 0.0001	2.28	1.79–2.92	< 0.0001	1.15	0.92–1.44	0.2154
	1–5	Ref.			Ref.			Ref.		

**Fig. 2:**
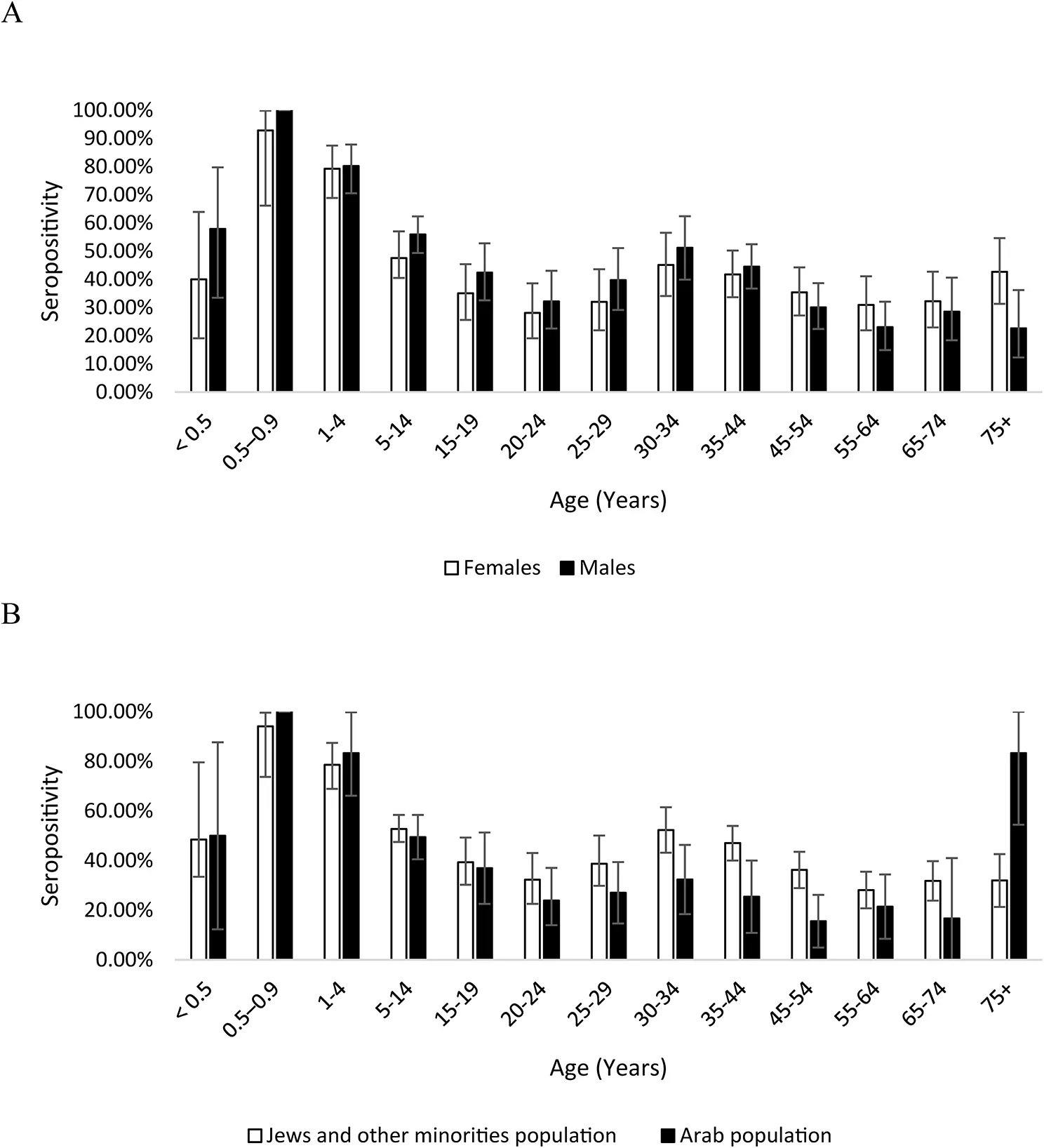
Prevalence of HBsAb detection in **A**- Males and females, **B**- Jews and other minorities population and Arab population

Following the observed interaction between sex and birth year, as well as significant differences identified among various ethnic and demographic groups in the study sample, we sought to explore in greater detail the distribution of HBsAb seropositivity by age and sex, and separately by age and ethnic group (Fig. 2). In the youngest age group (< 0.5 years), males exhibited higher HBsAb seropositivity (57.9%) compared to females (40.0%). This trend persisted across childhood and adolescence (e.g., 55.9% in males versus 47.6% in females for ages 5–14 years). However, starting from age 45 years and older, HBsAb seropositivity began reversing, with females demonstrating higher rates (e.g., 42.7% in females aged 75 and older compared to 22.6% in males). A similar pattern was observed across ethnic groups: while Jews and other minorities population exhibited higher HBsAb seropositivity in younger cohorts (e.g., 94.1% for 0.5–0.99 years vs. 100% in Arab population), this trend reversed in older age groups. By age 75 and older, Arab population showed significantly elevated HBsAb seropositivity at 83.3% compared to 32.0% in Jews and other minorities population. The ethnicity differences were statistically significant (*p* = 0.017). 

**Fig. 3:**
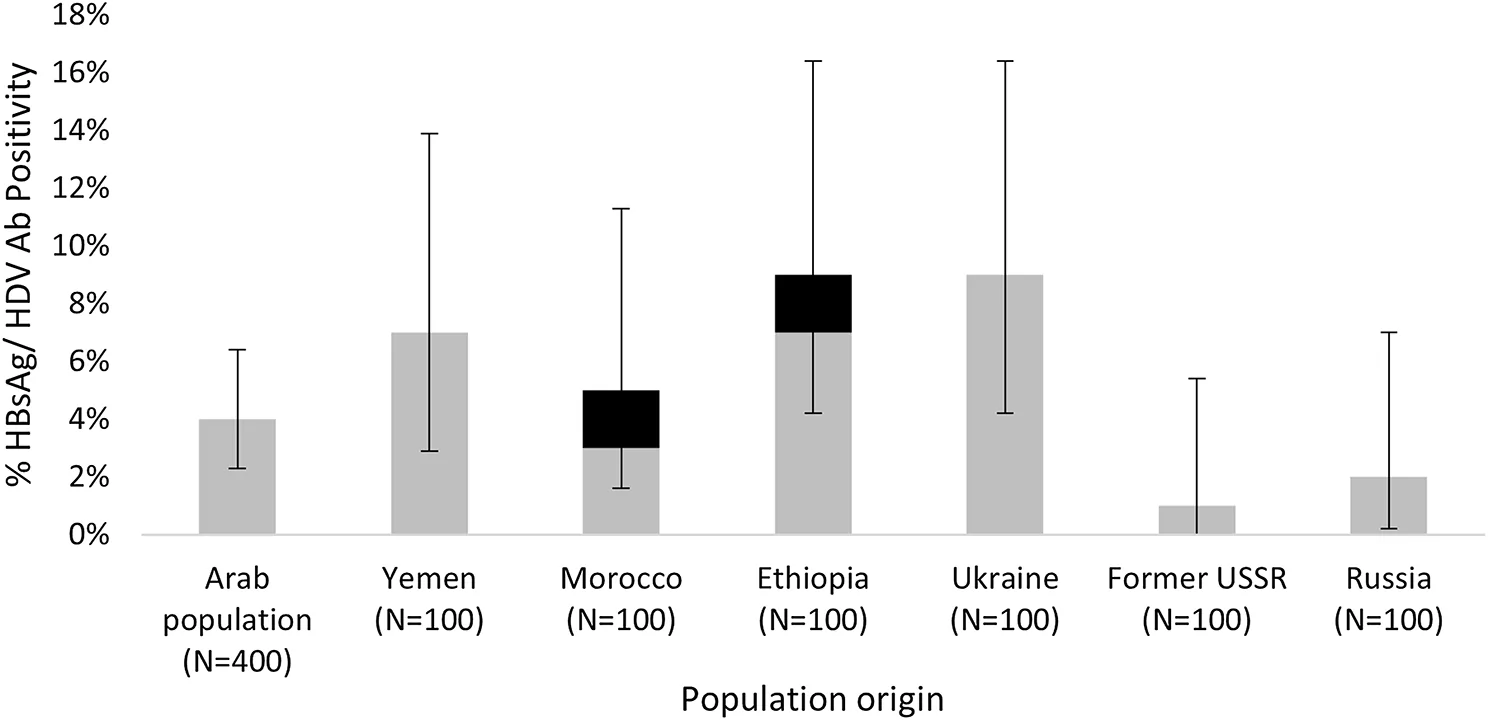
HBsAg and HDVAb seroprevalence among Israelis born before 1992, by country of origin. Grey- %HBsAg positivity. Black- %HBsAg/HDV-Ab positivity

Subsequently, HBsAg positivity was examined among population groups who immigrated to Israel from different countries of origin and were born before 1992. The distribution of HBsAg varied across different population groups (Fig. 3, p-value = 0.02). The proportion of positive individuals was highest among Israelis of Ethiopian (9%) and Ukrainian (9%) origin, followed by those of Yemenite (7%) and Moroccan (5%) origin. The lowest positivity rate was observed among Israelis of Arab (4%) and Former USSR origin (1.5%). Finally, out of the 49 HBsAg-positive samples from high-risk groups anti-HDV was tested, revealing 4 positives (8.2%), all from Morocco (*n* = 2) and Ethiopia (*n* = 2) (Fig. 3). Notably, all 4 were negative for HDV-RNA, suggesting resolved past exposure rather than active infection.

## Discussion

This nationwide serosurvey highlights the impact of Israel’s universal infant HBV vaccination program introduced in 1992, supporting its effectiveness while highlighting age-related waning of anti-HBs and historical gaps in vaccination coverage, as previously described [15]. This age-related gradient provides an important context for interpreting demographic disparities and is consistent with patterns observed in countries with long-standing vaccination programs. In Europe and other high-income settings, anti-HBs positivity exceeds 90% shortly after primary vaccination but declines to 30–60% by adolescence or early adulthood, reflecting the expected waning of antibody levels without necessarily indicating loss of clinical protection, owing to persistent immune memory [16, 17]. In Israel, the absence of an increase in acute HBV incidence or outbreaks despite these serologic trends is consistent with sustained population-level protection [18].

Regarding sex differences across birth cohorts, the enhanced immune response observed among older females may be attributed to sex-based immunological differences, including the stimulatory effects of estrogens and the suppressive effects of androgens on immune function [19]. Male sex is a significant predictor of nonresponse to hepatitis B vaccination, and males exhibit a steeper decline in antibody titers with age [20].

Ethnic differences were pronounced and cohort-dependent. The markedly higher HBsAb seropositivity observed among the oldest Arab population is consistent with historical exposure in Arab communities with intermediate endemicity prior to the introduction of vaccination [21]. These data suggest that higher rates of HBV infection and chronic carriage were present among the Arab population before the introduction of the vaccination program. Differences in access to healthcare services, particularly in historically underserved Arab communities, may have also contributed to these disparities [22], which were significantly narrowed following the introduction of the vaccination program [6].

Socioeconomic and geographic factors were also strongly associated with HBsAb seropositivity, with these differences driven primarily by pre-vaccination cohorts. Indeed, the implementation of a universal vaccination program has been shown to reduce disparities between socioeconomic groups [5].

Country of birth showed minimal impact on anti-HBs in the representative sample (Israel-born 42.9% vs. others 43.0%, non-significant), emphasizing age and SES as primary drivers. In contrast, among 1,000 high-risk pre-1992 samples, HBsAg carriage reached 9% in Ethiopian and Ukrainian, 5–7% in Yemenite/Moroccan, and lower in Arab population/ former USSR. These rates align with origin-country endemicity and European migrant data (3–9% HBsAg in high-risk groups vs. < 1–2% general population), underscoring targeted needs independent of population anti-HBs levels [23]. Targeted screening programs in such communities may be suggested, particularly for those born in high-endemic regions like Ethiopia and Ukraine. Similarly, the disparities observed among SES groups likely result from differences in healthcare infrastructure, access to public health information, and historical immunization coverage. Importantly, the diminished disparities in post-1992 cohorts underscore the success of the universal vaccination program, providing equitable access to vaccines as echoed in global studies, particularly in low-SES populations. Interestingly, the Arab population born before 1992 exhibited lower overall HBsAb seropositivity rates compared to Jews and other minorities populationHowever, their HBsAg carriage rates remained relatively low compared to other high-risk groups. This may be attributed to predominantly horizontal transmission of HBV in this population [24], resulting in lower rates of chronic HBV, rather than in vertical (perinatal) transmission [25]. Nonetheless, it appears that not all individuals fully cleared the virus or developed protective antibodies.

Finally, screening for HDV among the 49 HBsAg-positive high-risk individuals identified anti-HDV seropositivity in 4 cases (8.2%), exclusively from Moroccan and Ethiopian origins, aligning with historical endemicity in these regions [26, 27]. Indeed, a prevalence of 6.5% anti-HDV antibodies was identified in a cohort of nearly 9,000 HBsAg-positive individuals in Israel [28]. Here, the higher rate was served focusing on high-risk groups. The absence of HDV-RNA in all positives indicates resolved coinfection rather than active delta hepatitis, a favorable prognostic marker reducing immediate cirrhosis risk but warranting long-term monitoring. However, given the small sample size in this study, further validation in larger cohorts should be tested.

This study utilizes a cross-sectional design, and as such, the ability to infer causal relationships is inherently limited. Additional limitations include the wide confidence intervals for specific subgroups resulting from the limited number of identified HBsAg carriers. Finally, due to limited residual serum volumes and the broad scope of this population-level serosurvey, testing focused exclusively on our primary endpoints (HBsAb and HBsAg), preventing us from distinguishing vaccine-induced immunity from natural viral clearance, or detecting occult hepatitis B infection. Furthermore, due to the fully anonymized nature of the residual samples, we were unable to access clinical follow-up data or evaluate the rates of linkage to care and clinical interventions among the identified HBsAg carriers.

## Conclusions

In conclusion, Israel’s universal infant HBV vaccination program, introduced in 1992, has been highly effective in improving population-level protection, as demonstrated by the significantly higher HBsAb seropositivity among individuals born in or after 1992 compared with those born before 1992. Nevertheless, significant gaps remain. The identification of HBsAg carriage rates of up to 9% among specific adult immigrant populations from highly endemic regions highlights an important ongoing public health challenge. Maintaining the current infant vaccination strategy remains essential; however, it is unlikely to be sufficient on its own to address these remaining gaps. Policymakers should prioritize targeted education and screening programs for adult populations originating from highly endemic regions, coupled with effective linkage to care, appropriate clinical evaluation, and antiviral treatment when indicated. Addressing these specific gaps will be crucial for reducing HBV-related morbidity and contributing to the achievement of the WHO 2030 hepatitis B elimination targets.

## Supplementary Information

Below is the link to the electronic supplementary material.

Supplementary Material 1

## Data Availability

Data are available upon request from the authors.
