# Supplementary material for: National HBsAb serosurvey and HBsAg/Hepatitis D coinfection prevalence across sociodemographic and immigrant populations in Israel
Source: BMC Infect Dis. 2026 Sep 12;26:1933. doi: 10.1186/s12879-026-14403-2 (PMC13729391; doi:10.1186/s12879-026-14403-2)
Supplement: Supplementary file 1 [file 12879_2026_14403_MOESM1_ESM.docx]

Supplementary Table 1. Comparison of the study sample distribution and the Israeli general population by age group, sex, and population group

|  | **Sample population (ICDC)** | | | | **Israel population (Central Bureau of Statistics)** | | | |
| --- | --- | --- | --- | --- | --- | --- | --- | --- |
|  | **Jews and others** | | **Arabs** | | **Jews and others** | | **Arabs** | |
|  | **Males**  **N (%)** | **Females**  **N (%)** | **Males**  **N (%)** | **Females**  **N (%)** | **Males**  **N^*^ (%)** | **Females**  **N^*^ (%)** | **Males**  **N^*^ (%)** | **Females**  **N^*^ (%)** |
| **<1.00** | 24 (1.0) | 26 (1.1) | 3 (0.1) | 8 (0.3) | 33.5 (0.3) | 32.0 (0.3) | 10.0 (0.1) | 9.2 (0.1) |
| **1.00-4.99** | 67 (2.8) | 64 (2.6) | 24 (1.0) | 18 (0.7) | 287.3 (3.0) | 272.2 (2.8) | 88.6 (0.9) | 82.8 (0.9) |
| **5.00-14.99** | 189 (7.8) | 156 (6.4) | 47 (1.9) | 48 (2.0) | 696.0 (7.2) | 661.8 (6.9) | 211.3 (2.2) | 200.0 (2.1) |
| **15.00-19.99** | 77 (3.2) | 73 (3.0) | 22 (0.9) | 24 (1.0) | 293.0 (3.0) | 279.0 (2.9) | 101.3 (1.1) | 96.0 (1.0) |
| **20.00-24.99** | 65 (2.7) | 65 (2.7) | 22 (0.9) | 24 (1.0) | 262.1 (2.7) | 251.6 (2.6) | 101.8 (1.1) | 98.5 (1.0) |
| **25.00-29.99** | 64 (2.6) | 60 (2.5) | 19 (0.8) | 18 (0.7) | 237.8 (2.5) | 233.2 (2.4) | 89.0 (0.9) | 86.9 (0.9) |
| **30.00-34.99** | 65 (2.7) | 65 (2.7) | 17 (0.7) | 17 (0.7) | 231.4 (2.4) | 232.1 (2.4) | 78.5 (0.8) | 75.5 (0.8) |
| **35.00-44.99** | 135 (5.6) | 120 (5.0) | 29 (1.2) | 26 (1.0) | 457.1 (4.7) | 466.0 (4.8) | 121.4 (1.3) | 117.4 (1.2) |
| **45.00-54.99** | 109 (4.5) | 109 (4.5) | 24 (1.0) | 21 (0.9) | 411.4 (4.3) | 430.1 (4.5) | 107.1 (1.1) | 105.6 (1.1) |
| **55.00-64.99** | 76 (3.1) | 84 (3.5) | 15 (0.6) | 13 (0.5) | 319.2 (3.3) | 346.2 (3.6) | 73.1 (0.8) | 74.3 (0.8) |
| **65.00-74.99** | 65 (2.7) | 86 (3.5) | 5 (0.2) | 7 (0.3) | 291.5 (3.0) | 337.0 (3.5) | 36.1 (0.4) | 40.6 (0.4) |
| **75.00+** | 50 (2.1) | 72 (3.0) | 3 (0.1) | 3 (0.1) | 221.9 (2.3) | 301.8 (3.1) | 18.5 (0.2) | 24.1 (0.3) |
| **Total** | 986 (40.7) | 980 (40.4) | 230 (9.5) | 227 (9.4) | 3742.2 (38.8) | 3843.0 (39.9) | 1036.7 (10.8) | 1010.9 (10.5) |

* N thousands
